# Production of capsular polysaccharide does not influence *Staphylococcus aureus* vancomycin susceptibility

**DOI:** 10.1186/1471-2180-13-65

**Published:** 2013-03-22

**Authors:** Andrea Jansen, Christiane Szekat, Wiebke Schröder, Christiane Wolz, Christiane Goerke, Jean C Lee, Michael Türck, Gabriele Bierbaum

**Affiliations:** 1 Institut für Medizinische Mikrobiologie, Immunologie und Parasitologie, Universitätsklinikum Bonn, Sigmund-Freud-Str. 25, D-53105, Bonn, Germany; 2 Interfakultäres Institut für Mikrobiologie und Infektionsmedizin, Universität Tübingen,D-72076, Tübingen, Germany; 3 Channing Laboratory, Brigham and Women’s Hospital, Harvard Medical School, Boston 02115, USA

## Abstract

**Background:**

Diverse mechanisms (increased cell wall thickness, low cross linking, decreased autolysis, etc.) have been reported for *Staphylococcus aureus* strains with intermediate vancomycin susceptibility (VISA). This study was conducted to identify common mechanisms responsible for decreased vancomycin susceptibility in a VISA strain pair.

**Results:**

Transcriptional profiling of the clinical heterogeneous VISA isolate SA137/93A and its spontaneous homogeneous mutant strain SA137/93G pointed to an increased capsule production in the strain pair compared to a susceptible control. Furthermore, transcript quantification of the gene *cap5E*, which is essential for capsule biosynthesis, revealed elevated levels in the VISA strains SA137/93A, SA137/93G and Mu50 in comparison with susceptible strains Reynolds, Newman and SA1450/94. The increased expression was observed in bacteria from exponential as well as stationary growth phase. However, suppression of type 5 capsule formation by expression of antisense RNA did not increase vancomycin susceptibility in the VISA strain SA137/93G. Likewise, construction of inducible mutants of *S. aureus* Newman or repair of capsule biosynthesis of *S. aureus* HG001 and *S. aureus* 1450/94 did not influence resistance to vancomycin. Furthermore, purified type 5 polysaccharide did not protect indicator strains from the action of vancomycin.

**Conclusions:**

The VISA strain tested in this study displayed an increased production of type 5 capsular polysaccharide. However, the production of capsule material did not protect strain SA137/93G and three vancomycin sensitive strains in the presence of vancomycin and thus is not part of the resistance mechanism; however it may represent a by-product of VISA life style that is often characterized by a high sigma factor B activity.

## Background

The phenotype “intermediate vancomycin resistance” in *Staphylococcus aureus* (CLSI: MIC = 4–8 mg/L in Mueller Hinton broth (MH)) has been assigned to changes that lead to alterations in cell wall synthesis and morphology. Most vancomycin intermediately resistant *S. aureus* (VISA) strains are characterized by increased cell wall thickness as a consequence of activated cell wall biosynthesis and decreased autolysis [1-7]. The mechanism of resistance was shown to be based on an enhanced amount of free d-Ala-d-Ala termini in the cell wall, which act as false target sites that keep the vancomycin molecules from reaching lipid II [2,8]. Many studies have attempted to elucidate the genetic basis of this resistance type, mainly by comparative transcriptional profiling and full genome sequencing (for a review see [9]). Transcriptional profiling revealed a variety of genes that were differentially expressed in VISA strains or resistant laboratory mutants (MIC: ≥ 32 mg/L) in comparison with vancomycin susceptible *S. aureus* (VSSA). From these results it was postulated that an activated sugar and lipid metabolism and increased energy are required to generate thicker cell walls in VISA strains [10-12]. Furthermore, mutations in two component regulatory systems (*yycFG*, which was recently renamed *walKR*, *yvqF*/*vraSR* and *graRS*) are assumed to play a central role in adaptation to the antibiotic stress [9,13-19], as well as mutations in *rpoB *[20-22], *pknB *[23], *prsA *[24] and *clpP *[25].

The clinical methicillin resistant VISA isolate SA137/93A was isolated from a tracheal secretion and displays heterogeneous intermediate vancomycin resistance (hVISA strain, MIC: 2 mg/L in MH, 8 mg/L in brain heart infusion (BHI)). Subculturing in the presence of 6 mg/L vancomycin generated a mutant with homogeneous intermediate vancomycin resistance, which showed an MIC value of 16 mg/L in BHI (4 mg/L in MH) and was designated SA137/93G [4]. Pulsed-field gel electrophoresis (PFGE) profiles, phage typing and MLST sequencing of the strains showed that they were members of the Iberian clone (ST247) which was prevalent in Germany in the early 1990’s under the designation “Northern German epidemic strain”. Both strains possess a thickened cell wall [4]. The decreased vancomycin susceptibility of strain SA137/93A is most probably based on an increased amount of free d-Ala-d-Ala termini in the cell wall, which is due to decreased crosslinking. Surprisingly, the cell wall cross linking of strain SA137/93G was within the standard range [4]. As a first step in analysis of the genetic background of the decreased vancomycin susceptibility of both strains, the insertion patterns of the highly mobile insertion element IS*256* were compared and found to be different. Strain SA137/93G is characterized by an insertion of IS*256* into the gene *tcaA *[26,27] and reconstitution of *tcaA* led to a decrease in vancomycin resistance. In contrast, strain SA137/93A displays an IS*256* insertion in the promoter region of the essential two-component system *yycFG (walRK)* which leads to an increased expression of this system [27]. However, although both insertions were shown to correlate with a decrease in susceptibility to vancomycin, the difference in the vancomycin resistance level of the strain pair could be mainly attributed to the disruption of *tcaA* in SA137/93G [27]. Furthermore, SA137/93G carries a deletion which starts at the IS*431* element at the left junction of the SCCmec and covers a chromosomal fragment that comprises SA0027 to SA0132 [4]. Similar deletions starting at the very same bp have been described for MRSA strains after storage in the laboratory [28]. The absence of *mecA* also contributed to the higher vancomycin resistance of strain SA137/93G [4].

This study was conducted to identify common mechanisms responsible for decreased vancomycin susceptibility in the hVISA isolate SA137/93A and its homogeneous resistant derivative SA137/93G. To this end, we compared the transcriptomes of both strains with that of the closely related vancomycin susceptible MRSA strain SA1450/94 (MIC: 2 mg/L in BHI). We found that the genes encoding capsule biosynthesis were the only genes with higher expression in both VISA strains and therefore tested whether the staphylococcal type 5 capsular polysaccharide (CP5) might be involved in vancomycin resistance of this strain.

## Methods

### Bacterial strains, plasmids, growth conditions and antimicrobial susceptibility testing

Bacterial strains and plasmids are listed in Table 1. *S. aureus* strains were cultured in BHI medium, containing 2 g/L glucose (Becton Dickinson GmbH, Heidelberg, Germany) at 37°C with aeration, unless indicated otherwise. For every experiment an overnight culture was diluted 100-fold in fresh BHI broth and further incubated to an optical density at 600 nm (OD_600_) of 0.8-1.0 to ensure exponential growth conditions. Determination of vancomycin MICs was performed following the microdilution method according to CLSI guidelines except using BHI medium unless indicated otherwise. For E-testing cultures were diluted to an optical density of 2 McFarland and plated on BHI agar. Population analysis was carried out as described previously [29]. Some antisense experiments were performed on TSA without glucose (TSA-G). Antisense plasmids were selected with 34 μg/ml chloramphenicol (CM) as described for the system [30]; otherwise 20 μg/ml of chloramphenicol was employed.

**Table 1:** Strains and plasmids

**Strain/plasmid**	**Relevant genotype or phenotype**^**a**^	**Source/reference**
***S. aureus *strains**		
SA137/93A	Clinical hVISA isolate; MET^r^, Northern German epidemic MRSA, MLST sequence type ST247	[4]
SA137/93G	Mutant VISA of SA137/93A; Δ*SCCmec* (MET^s^), ST247	[4,27]
SA137/93G pCapDvorne	SA137/93G carrying pCapDvorne (CHL^r^)	This work
Mu50	Clinical VISA isolate	[29]
SA1450/94	Northern German epidemic MRSA, Iberian clone, ST247, CP5 positive, VAN^s^ control	German reference centre for staphylococci, Wernigerode
SA1450/94 pCapAre	SA1450/94, repair of *cap5A*	This work
SA1450/94 pCU1	SA1450/94, control (empty vector)	This work
Newman	CP5 positive, VAN^s^	NCTC8178
Newman-132	pMUTIN integrated into capsule promoter	This work
NCTC8325 (RN1)	laboratory strain	National collection of type cultures
HG001	NCTC8325, repaired in *rsbU*	[31]
HG001 pCap5E	HG001 capsule production repaired	This work
SG511-Berlin	sensitive antibiotic screening strain	[32]
RN4220	Restriction negative derivative of NCTC8325, cloning host	[33]
**Plasmids**		
pUC19*gyrB*	pUC19, carrying a 560 bp internal fragment of *gyrB*; external plasmid standard for real time PCR	This work
pCU1*cap5E*	pCU1 (Augustin), carrying *cap5E*; external plasmid standard for real time PCR	This work
pCapDvorne	pEPSA5 (Forsythe) harbouring a *cap5D* antisense fragment	This work
pCapAre	pCU1 harbouring *cap5A*	[34]
pCap5E	pCU1 harbouring *cap5E*	[34]
pCG132	pMUTIN4 (Vagner 1998) habouring *cap5A*	This work

^a^Abbreviations: *CP*, capsular polysaccharides; *CHL*, chloramphenicol; *MET*, methicillin; *VAN*, vancomycin; r, resistant; s, susceptible.

### Comparative transcriptomics

For transcriptional profiling, the strains compared were grown to an OD_600_ of 0.8-1.0. Preparation of total RNA, cDNA synthesis and fluorescence labelling as well as microarray experiments using the *sciTRACER S. aureus* N315 full genome chip (Scienion AG, Berlin, Germany) were performed as described previously [27]. The respective experiments were replicated at least four times including a dye swap. The microarray data were deposited in the gene expression omnibus (GEO) database at NCBI under accession number GSE10529.

### Comparative genomics

Genomic DNA of the strains SA137/93A, SA137/93G and SA1450/94 was prepared employing genomic tip 20 columns (Qiagen, Hilden, Germany) according to the manufacturer’s instructions. Cell lysis was supported by incubating the cell suspension for 1 h at 37°C in the presence of 50 mg/L lysostaphin. Genomic DNA (3 μg) was labelled using the Bioprime DNA labelling system (Invitrogen, Karlsruhe, Germany) following the instruction manual. The labelling reaction was performed in the presence of 0.1 mM cyanine-3’- or cyanine-5’-labelled dCTP (Perkin Elmer Life Science, Mechelen, Belgium) in addition to 0.2 mM dCTP, 0.5 mM dATP, 0.5 mM dGTP and 0.5 mM TTP. The labelled DNA was purified using the MinElute purification kit (Qiagen) and subsequently compared by competitive hybridisation employing the *sciTRACER S. aureus* N315 full genome chip as described previously [27]. The experiment was conducted in duplicate including a dye swap.

### Immunofluorescence labelling of CP5

The incubation time and media employed for capsule production are indicated in the figure legends. CP5 production was detected by an indirect immunofluorescence technique [35]. In brief, bacteria were fixed to microscope slides with heat and incubated for one hour with human serum to saturate protein A. The human serum had been pretreated with protein A deficient strain Newman (diluted 1:10 in PBS with 0.05% Tween 20) to remove existing *S. aureus* antibodies from the serum. Slides were washed and incubated for 1 h at ambient temperature with rabbit antiserum specific for CP5 and diluted 1:200 in PBS with 0.05% Tween 20. The slides were again washed three times before incubation with CY3-conjugated goat F(ab)_2_ fragments raised to rabbit IgG (Dianova, Hamburg, Germany) diluted 1:500 in PBS with 0.05% Tween 20. In a subsequent step, the bacteria were stained with 4,6-diamidino-2-phenylindol (DAPI, 2 mg/L; Sigma-Aldrich, Munich, Germany) for 5 min at room temperature.

### Transcript quantification by real time PCR

Cells of the VISA strains SA137/93A and SA137/93G and the susceptible controls SA1450/94 and Newman (the CP5 type strain) were harvested from a culture at OD_600_ 0.3, 0.5, 1, 2 and 4–5. RNA preparation and cDNA synthesis were done as previously described [27]. Experiments were conducted at least in duplicate for each strain. Transcript amounts of *cap5E* were determined in LightCycler (Roche Diagnostics, Mannheim, Germany) experiments by quantification relative to the housekeeping gene *gyrB* employing external plasmid standards (Table 1) as described previously [27]. PCR experiments were conducted using the LightCycler FastStart DNA Master SYBR Green I Kit (Roche Diagnostics, Mannheim, Germany) according to the manufacturer’s instructions and the gene specific primer pairs gyrB-1-RT and gyrB-2-RT [27] and cap5E-1-RT (CCAGTTGAGGCAGTGAAGACA; NCBI: NC_002745 bp 171655–676) and cap5E-2-RT (CTGATCCTCTTGAAGCCATCAC; NCBI: NC_002745 bp 171878–899), respectively. The following temperature profile was utilized for amplification: Initial denaturation at 95°C for 10 minutes (20°C/s). 45 cycles of denaturation (95°C; 1 s; 20°C/s), annealing (55°C; 15 s; 20°C/s), elongation (72°C; 15 s; 20°C/s; single mode). Specificity of the PCR reaction was verified by melting curve analysis (45°C (10 s; 20°C/s) to 95°C (0.2°C/s), continuous mode) and ethidium bromide staining on agarose gels. Calculation was done by the second-derivative maximum method. The quantification assays were conducted employing RNA prepared from two independent cultures of each strain.

### Antisense experiments

A 166 bp fragment located in the N-terminus of *cap5D* was amplified using the primers capD-vorne-166_anti-for (AAATCTAGAATCTGTGAAATTGCGGCTTT) and capD-vorne-166_anti-rev (AAAGAATTCTGCTGAAATATGATGCGATATG) with Phusion DNA polymerase (New England Biolabs, Frankfurt, Germany) and ligated to the vector pEPSA5 [30] using the *Xba*I and *Eco*RI restriction sites. The ligation assay was transformed into *E. coli* JM83 by electroporation, the recombinant plasmid was shuttled into *S. aureus* RN4220 by electroporation [36] and subsequently transduced into *S. aureus* SA137/93G by phage transduction using bacteriophage 80α [37]. For expression of antisense RNA, the cultures were grown in LB (lysogeny broth)/CM34 or other media as indicated [30] and were divided for addition of 50 mM xylose to one of the cultures. Sequencing confirmed that pEPSA5 does not contain the *cre* sequence, which would inhibit transcription in the presence of glucose.

### Complementation of *cap5E*

The defect in Cap5E in strains of the NCTC 8325 lineage (the M134R exchange that leads to inactivation of the protein) was complemented using *cap5E* on pCU1 as described in [34]. The DNA fragment harbouring *cap5E* (bp 3394–5448 in NCBI acc. nr. U81973, [34]) was amplified by PCR employing the primers cap5Eforward (GCTTCTAGACTAGTTTTGCAGGCAGG) and cap5Ereverse (GTCGAGCTCGTTAAATCTGCTTTCAA) from *S. aureus* Newman DNA, ligated into pCU1 and after subcloning in *E. coli* and *S. aureus* RN4220 the recombinant plasmid was introduced into *S. aureus* HG001 [31].

### Generation of a conditional capsule mutant

In gram-positive bacteria, pMUTIN4 is an integrative vector that places the downstream genes under control of a Pspac promoter [38]. The *cap5A* gene was amplified using the primers EcoRIcap431-for (GGGGGAATTCTAAGGGAGGTAAATAATGG) and BamHcap1431-rev (ACACGGATCCATTAAGCTTGATAGTCCA), ligated with the restricted (*Eco*RI, *Bam*HI) pMUTIN4 plasmid and transformed into *E. coli*. The resulting plasmid (pCG132) was verified by sequencing and electroporated into *S. aureus* strain RN4220. Since pMUTIN4 does not have a gram-positive origin of replication, all clones had gone through a single crossover event, which inserted the vector into the genome and placed the *cap5A* gene under the control of the IPTG-inducible Pspac promoter. The integrated plasmid was then transduced into strain Newman using Φ11 lysates. Mutants were verified by PCR using the oligonucleotides P5spac (TACATCCAGAACAACCTCTG) and capArev (GACTTTAACTGCTGTACCGTCTGCT) and PFGE.

### Extraction of capsular polysaccharides (CP)

For extraction of crude capsule extract, staphylococci were plated onto Columbia blood agar plates that had been supplemented with 50 mM NaCl. After 24 h of incubation at 37°C, the bacteria were harvested by suspension in PBS buffer. The CP was detached from the cells by autoclaving at 120°C for 1 h and the cell debris was removed by centrifugation. The supernatant was passed through a cellulose acetate filter (pore size 0.45 μm). Cell wall teichoic acid was removed by treatment with 50 mM NaIO_4_ for 72 h at room temperature in the dark [39]. The crude extract was then washed with PBS buffer by ultrafiltration on a YM10 membrane (Millipore, Schwalbach, Germany) or employing Vivaspin 6 columns (exclusion volume of 3 kDa) (Sartorius, Göttingen Germany). These extracts were then added to MIC determinations in MH medium using *S. aureus* NCTC 8325 and *S. aureus* SG511 as indicator strains. In order to test for contaminating nucleic acids, the extracts were digested with DNase and RNAse [40] and tested again. Crude capsule extract from *S. aureus* NCTC 8325 which cannot produce a capsule because of the point mutation in Cap5E and PBS buffer served as negative controls in these experiments. Purified CP5 was obtained as described in [41].

### Sequencing of the promoter region of the CP5 biosynthesis gene cluster

A 735 bp DNA segment comprising the promoter region of the CP5 biosynthesis gene cluster was amplified using a standard PCR protocol and the primer pair (AGCTCGCATTTGAAGATCAATGT) and (CCTCTTGTGCCATAAACTGAGG) (bp 166966–166988 and bp 167586–167607, NCBI: NC_002745). The product was purified (QIAquick Gel Extraction Kit, Qiagen, Hilden, Germany) and sequenced (Sequiserve, Vaterstetten, Germany).

Detection of the *cap5* gene cluster in the VISA strains was performed using primers  cap5-9864 (GTACGAAGCGTTTTGATAGTT) and cap5-9332 (GAAAGTGAACGATTAGTAGAA) that flank the type-specific sequences of *cap5I* and *cap5J* in *S. aureus *[42].

The insertion of IS*256* in *cap5A* in *S. aureus* SA1450/94 was complemented by reconstituting *cap5A* on the plasmid pCapAre, exactly as described in [34]. The fragment was amplified employing genomic DNA of *S. aureus* SA137/93G as a template and the primers pCapAreconfor (GCAGAGCTCGCATTTGAA) and pCapAreconrev (CCAATGATTAAGCTTGATAGTCC). These primers harbour the natural cleavage sites that had been used for complementation previously [34].

## Results and discussion

### Comparative transcriptomics

The clinical VISA isolate SA137/93A and the type strain of the Iberian clone in Germany (‘Northern German epidemic strain’) SA1450/94 showed identical PFGE patterns [43] and MLST types (ST247). In order to further confirm that SA1450/94 is a suitable control strain, chromosomal DNA of SA137/93A was competitively hybridised to that of SA1450/94 and SA137/93G, respectively. The microarray results showed that all ORFs present in the VISA strain SA137/93A were also present in strain SA1450/94. In addition, the competitive hybridisation precisely reflected the deletion in the mutant SA137/93G [4].

Comparative transcriptomics of the hVISA isolate SA137/93A and SA1450/94 revealed that there were only 15 genes showing a higher expression level in the hVISA strain (2- to12-fold; see Additional file 1: gene expression data.pdf, Additional file 1: Table S1). The *yycFGHI*-operon [27] and three genes of the type 5/8 capsule biosynthesis gene cluster (*capB*, *capC*, *capE*), which showed a 4- to 5-fold higher expression, were among the ten genes with a known function in Additional file 1: Table S1. The relatively low number of regulated genes may be due to the fact that the strain shows a heterogeneous phenotype, i.e., only a subpopulation of the culture displays high resistance to vancomycin. Similar results were obtained with the JH series of mutants; here JH1 to JH5 did not show any alterations in gene expression, although resistance had increased [44,45], therefore, this observation was not surprising.

**Figure 1:**
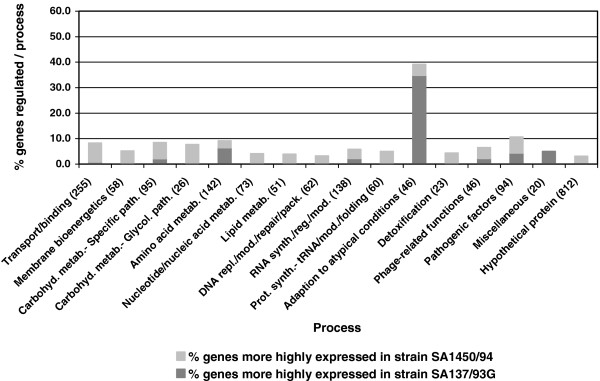
**Transcription profiling: comparison of transcriptomes (OD**_**600 **_**= 0.8-1.0) of VISA strain SA137/93G and the related VSSA strain SA1450/94.** The regulated genes are represented as percentage of all genes constituting a process category. The number of genes per process category is shown in brackets.

As expected, the transcription profile of the VISA strain SA137/93G differed more strikingly from that of SA1450/94. A total of 124 genes showed at least a twofold change in strain SA137/93G (2- to 13.7-fold; see Additional file 1: gene expression data.pdf, Additional file 1: Table S2) compared to SA1450/94. 30.6% of these genes encoded hypothetical proteins. Figure 1 shows the percentage of regulated genes in the different functional gene classes. Only one category of genes, “adaptation to atypical conditions”, which comprises genes encoding capsule biosynthesis enzymes, chaperones, heat and cold shock proteins and the *clp* protease, was overrepresented among the genes showing higher transcription levels. The 16 genes of the capsular biosynthesis gene cluster *capABCDEFGHIJKLMNOP* were - on average - six fold up-regulated. Additionally, a more than twofold increase in transcript amounts was found for the gene encoding AlsS, which is involved in formation of acetoine from pyruvate and influences the regulation of autolysis [46], the urease operon and two ORFs of the *ica* gene cluster. All of the above mentioned genes have also been found to be up-regulated under mildly acidic conditions [47]. Since some of the genes with lower expression levels in strain SA137/93G were also down-regulated under acidic conditions, part of the expression profile might be the consequence of differences in the pH decrease of the glucose-containing BHI medium that was used for cultivating the strains. This hypothesis is supported by the finding of Nelson *et al.*[48] indicating that an impaired catabolism of acetate seems to be typical for some VISA strains and might result in the up-regulation of urease, which supplies ammonium ions that neutralize the decrease in pH caused by the formation of acids [49]. In addition, the capsule gene cluster, *alsS* and SA2262, SA2367 as well as SA2403 are members of the sigB regulon and might indicate an increased SigB activity which has been shown to contribute towards glycopeptide resistance [50]. A more than twofold decrease in expression was observed for 80 genes (2- to 13.7-fold) in the VISA strain SA137/93G in comparison with the susceptible control. In summary, an increased transcription of genes involved in capsule biosynthesis was the only expression pattern that was common to both VISA strains in comparison to the VSSA strain.

### *Cap5E* transcript quantification by real time PCR

**Figure 2:**
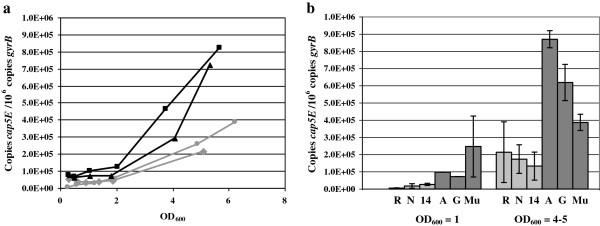
**Transcript quantification of the essential capsule biosynthesis gene *cap5E *by real time PCR. a**) Transcript amounts of *cap5E* throughout the growth curve of hVISA SA137/93A (filled square), VISA SA137/93G (filled triangle), VSSA Newman (filled circle) and VSSA SA1450/94 (filled diamond) indicated as copy number per 10^6^ copies of the housekeeping gene *gyrB*. **b**) Transcript amounts of *cap5E* of VSSA strains (R: Reynolds*, N: Newman, 14: SA1450/94) and VISA strains (A: SA137/93A*, G: SA137/93G*, Mu: Mu50) at OD_600_ = 1 and OD_600_ = 4–5 indicated as copy number per 10^6^ copies of *gyrB*. * Error bars are not visible at OD_600_ = 1 because of minimal data variations.

The *cap5* and the *cap8* loci are allelic, each comprising 16 genes (*capA*-*P*) that are transcribed in one orientation with 12 of the 16 genes being nearly identical. The four genes in the central region of the cluster are type-specific and show little homology [51]. The presence of the type 5 gene cluster in the VISA strains and SA1450/94 had been indicated by the microarray results and was confirmed by PCR. In *S. aureus*, capsule production occurs primarily in the late log and post-exponential growth phase. It had previously been shown that *S. aureus* CPs are not detectable before the late log growth phase, 2 h after the transcript increase in the mid log phase [52,53]. For exact quantitative analysis of expression of the CP biosynthetic enzymes and to obtain further insights into capsule production in different growth phases, the transcription level of the essential capsule gene *cap5E *[34] was determined by real time PCR. Figure 2a shows the expression rate of *cap5E* throughout the growth curve of the VISA strains and the controls. The expression patterns during growth were similar in all tested strains. A strong increase of capsule expression occurred in the post-exponential growth phase after the culture reached an optical density of 2 (Figure 2a) in VSSA and VISA strains, and the basal expression level in strain SA137/93A and SA137/93G was already elevated during the early growth phase. Furthermore, an increase of *cap5E* gene transcription could be observed in the stationary growth phase in the VISA strains, with a 2- to 3-fold increased expression level at an OD_600_ of about 5. A lower transcription in VSSA strains in exponential and stationary phase could be confirmed when strain Reynolds and Newman were included into the measurements (Figure 2b). These findings were consistent with the primary microarray-based data of this study and expression data of *S. aureus* Mu50 [10].

The CPs of *Klebsiella pneumoniae* were found to contribute to resistance to cationic defensins, lactoferrin, protamine sulfate and polymyxin B, and in this context, the capsule was assumed to protect bacteria by limiting the interaction of the antimicrobial peptides with the surface [54]. Later, similar results were obtained with polysaccharides from *Streptococcus pneumoniae* and alginate from *Pseudomonas aeruginosa *[55]. A possible role of CPs in vancomycin resistance has repeatedly been discussed in the literature. Boyle-Vavra *et al.* found that susceptible passage revertants of the CP5 producing VISA isolates MI, NJ and PC were no longer CP typable, while passaging in presence of vancomycin retained the CP phenotype [56]. Besides, comparative expression profiling experiments on VISA isolates Mu50, MI, JH9 and their respective susceptible parent or mutant strains showed that some (but not necessarily all) of the genes of the type 5 capsule were more highly expressed in the VISA strains [10,45]. Enhanced capsule production in other VISA was also reported [57] and deletion of the *yabJ-spoVG* operon affected glycopeptide susceptibility and capsule production in *S. aureus* simultaneously [50]. Taken together, these findings encouraged us to further investigate the role of CPs in vancomycin resistance.

### Detection of the capsule by immunofluorescence

**Figure 3:**
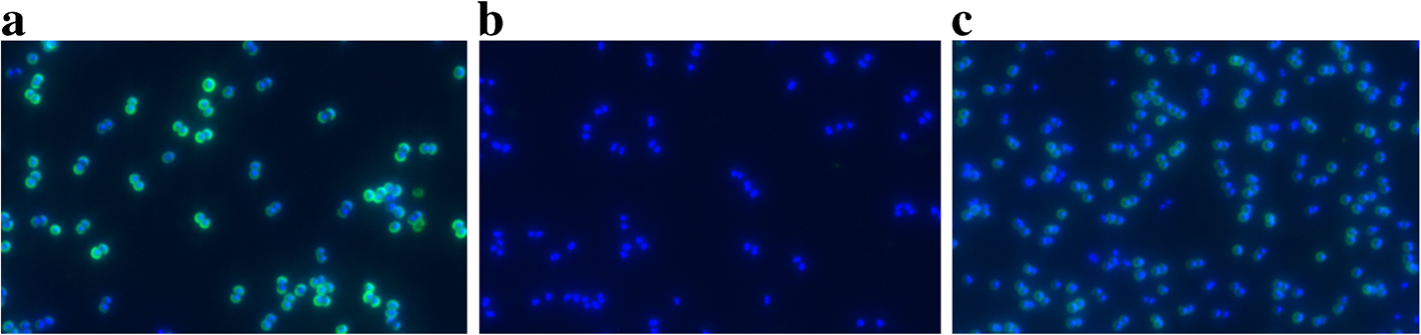
**Comparison of CP5 production in a VISA and two VSSA strains.** CP5 was labelled by immunofluorescence (CY3, green). As a control, all cells were stained using DAPI (blue). Cells were grown for 6 h in LB at 37°C. **a**) VISA SA137/93G; **b**) control strain SA1450/94; **c**) *S. aureus* Newman.

Production of CP5 was analysed by immunofluorescent labelling of cells of SA137/93G and the susceptible strains SA1450/94 and Newman after 6 h of incubation in LB. The results revealed that the VISA strain produced higher amounts of CP5 than SA1450/94 and *S. aureus* Newman (Figure 3).

### Sequence of the capsule promoter of VISA strains and SA1450/94

In order to detect possible changes in the promoter of the capsule gene cluster that might be responsible for an elevated expression in the VISA strains, a region covering 400 bp upstream of *cap5A* was sequenced. The complete sequences were identical to that published for *S. aureus* COL (ST250), which is a close relative of the Iberian strain, and *S. aureus* RF122. The promoter sequence of the *cap5* gene cluster and the inverted repeats that constitute the operator [58,59] were identical to that of the first seven published genomes. Unexpectedly, the control strain SA1450/94 showed an insertion of IS*256* into the first gene of the capsule gene cluster *cap5A1.* The IS element was located 50 bp downstream of the ATG start codon and oriented in an antisense direction. Cap5A1 encodes a membrane protein that is part of the protein kinase Cap5A1/Cap5B2, which is needed for phosphorylation of Cap5O [60]. In spite of this, in in vitro experiments Cap5A1 is not essential for activation of Cap5O since a paralogue of Cap5A1, Cap5A2 is encoded by SA2457 and able to activate the kinase subunit Cap5B2 [60]; this is also demonstrated by the fact that SA1450/94 was able to produce capsule, albeit at low levels, in overnight cultures (data not shown).

### The effect of capsule on vancomycin resistance in VISA

**Figure 4:**
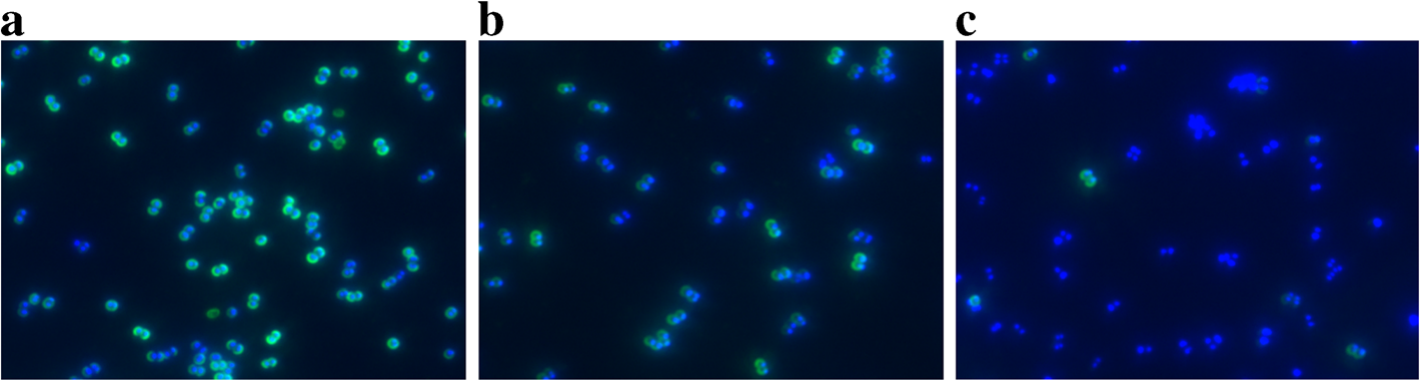
**Suppression of capsule formation by expression of *cap5D*-antisense RNA.** CP5 was labelled by immunofluorescence (CY3, green), the cells were stained using DAPI (blue). Cells were grown for 6 h in LB at 37°C. **a**) *S. aureus* SA137/93G (control); **b**) *S. aureus* SA137/93G harbouring pCapDvorne in the absence of xylose and **c**) grown in the presence of 50 mM xylose.

Initial attempts to knock out capsule production in the VISA strains resulted in mutants that could not be complemented because they harboured background mutations in regulatory genes that are necessary for capsule production and influence glycopeptide susceptibility (*rsbU, agr*), e.g., inactivation of *rsbU* led to an increase in vancomycin susceptibility in our isolates even if capsule biosynthesis had been reconstituted. Therefore, we chose an antisense approach. An N-terminal 166 bp fragment of *cap5D* was ligated to pEPSA5 in antisense direction and transformed into *S. aureus* 137/93G. We chose another region than that described in [30] since antisense RNA expression from this fragment had exerted growth-inhibitory effects. Capsule formation was analyzed by immunofluorescence in the absence and presence of 50 mM xylose in different media (LB, BHI and CYPG [61]) after 6 h of incubation. Figure 4 shows that after only 6 h of incubation, capsule formation in the wildtype SA137/93G is relatively strong even in LB (Figure 4c), and that the capsule formation is somewhat decreased in the presence of the plasmid even in the absence of xylose (Figure 4b). Addition of 50 mM xylose (but not 12.5 mM) led to a full repression of capsule biosynthesis (Figure 4c) in all tested media with the exception of a few cells that had obviously been able to eliminate the plasmid.

In MIC determinations in LB/CM 34, no significant difference in vancomycin resistance was observed after expression of antisense RNA in *S. aureus* SA137/93G. The value of 1.5 ± 0.4 mg/L vancomycin obtained for encapsulated strains grown in the absence of xylose was lowered to 1.3 ± 0.3 mg/L vancomycin for capsule-free cells incubated in the presence of xylose.

**Figure 5:**
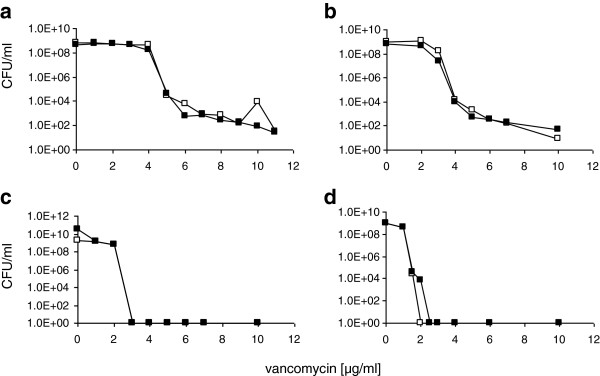
**Population analyses of different strains in the presence or absence of capsule. a**) *S. aureus* SA137/93G harbouring pCapDvorne grown on BHI agar in the absence of xylose (capsule; □ ) or in the presence of xylose (no capsule; ▄ ); **b**) *S. aureus* SA137/93G harbouring pCapDvorne grown on TSA without glucose in the absence (□ ) or in the presence of xylose (▄ ); **c**) *S. aureus* HG001 (□ ) and *S. aureus* HG001 harbouring pcap5E (▄ ) which leads to reconstitution of capsule biosynthesis on BHI agar; **d**) *S. aureus* Newman harbouring an insertion of pMUTIN4 in the capsule promoter grown on MH agar in the absence (□ ) and the presence (▄ ) of 1 mM IPTG.

Intermediate vancomycin susceptibility of VISA strains is most easily demonstrated in population analyses on BHI, which is the medium that yields the highest vancomycin MICs and therefore should be the most sensitive medium. Again there was no difference in the population analyses of clones grown in the absence or presence of xylose (Figure 5a). Experiments in TSA-G (TSA without glucose) yielded similar results (Figure 5b).

### The effect of the capsule on vancomycin resistance in VSSA

**Figure 6:**
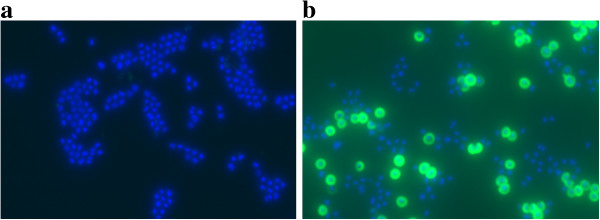
**Repair of capsule formation in *S. aureus *HG001.** CP5 was labelled by immunofluorescence (CY3, green), the cells were stained using DAPI (blue). Cells were grown in TSB medium overnight at 37°C. **a**) *S. aureus* HG001 (control); **b**) *S. aureus* HG001 pCap5E, in which capsule production has been reconstituted.

In addition to the VISA strain, the effect of the capsule on vancomycin resistance in three vancomycin susceptible strains producing CP5 was investigated. All strains of the RN1 (NCTC 8325) lineage harbour a mutation in *cap5E* that leads to inactivation of capsule biosynthesis. Furthermore a deletion in *rsbU* leads to a very low activity of sigma B which however is needed for the efficient transcription of the capsule biosynthetic genes [50]. As described in [34], capsule production was reconstituted into *S. aureus* HG001 (*rsbU* repaired) by introduction of a plasmid carrying a *cap5E* gene amplified from *S. aureus* Newman (Figure 6). Again the population showed a heterogeneous phenotype in immunofluorescence experiments. However, in population analyses no increase in resistance against vancomycin could be detected (Figure 5c).

**Figure 7:**
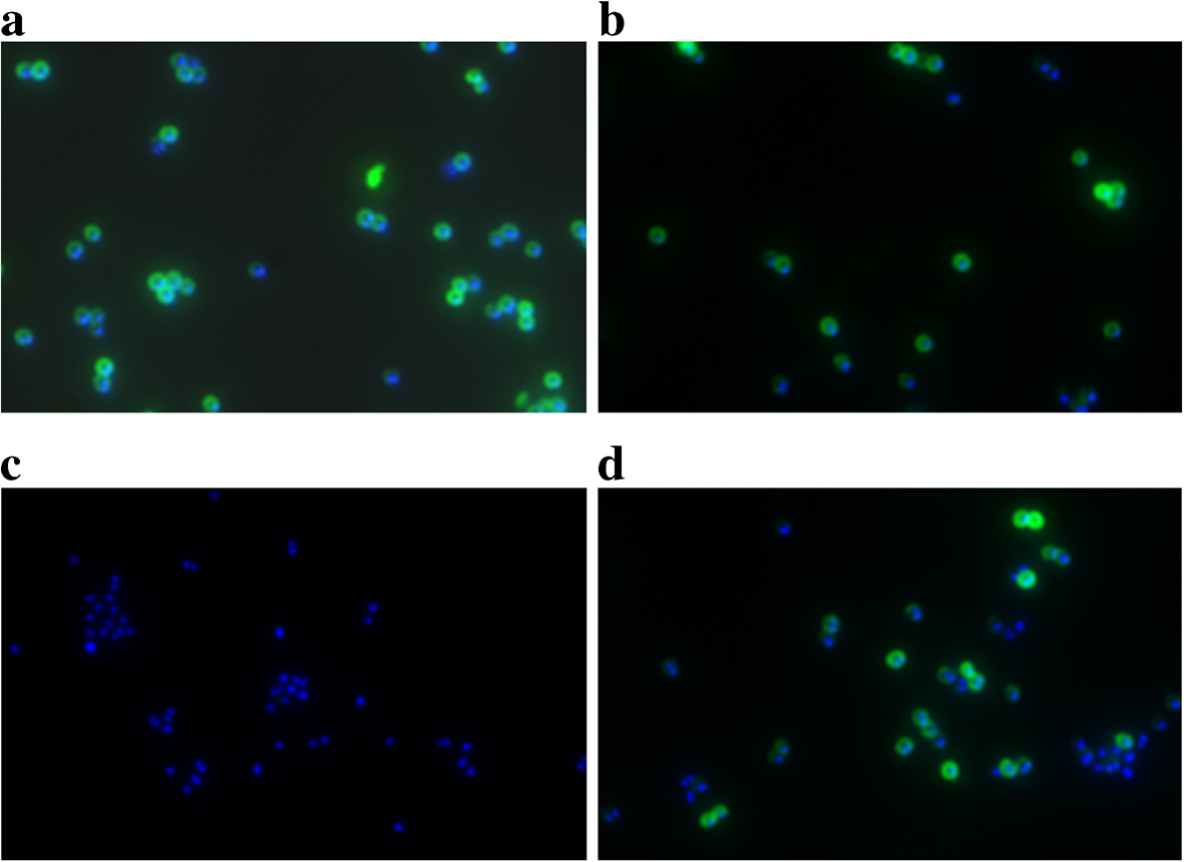
**Induction of capsule production by IPTG in *S. aureus *Newman-132.** CP5 was labelled by immunofluorescence (CY3, green), the cells were stained using DAPI (blue). Cells were grown for 6 h in MH medium at 37°C. **a**) *S. aureus* Newman (control) **b**) *S. aureus* Newman in the presence of 0.5 mM IPTG; **c**) *S. aureus* Newman-132 harbouring pMUTIN4 in the capsule promoter in the absence of IPTG and **d**) *S. aureus* Newman-132 harbouring pMUTIN4 in the capsule promoter after induction with IPTG.

An *S. aureus* Newman clone with the capsule promoter under control of Pspac was capsule negative in the absence of inducer, but heterogeneous capsule production could be achieved by addition of IPTG to media that did not contain glucose, e.g., MH (Figure 7). In population analyses with these cells, no significant difference in vancomycin resistance on MH agar was visible (Figure 5d).

**Figure 8:**
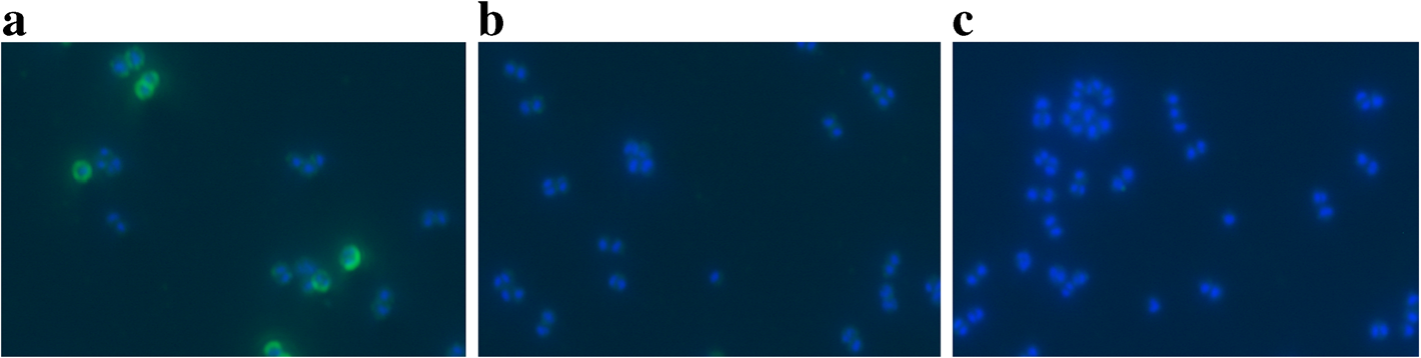
**Capsule production of different *S. aureus *SA1450/94 clones.** CP5 was labelled by immunofluorescence (CY3, green), the cells were stained using DAPI (blue). Cells were grown for 6 hours in BHI medium at 37°C. **a**) *S. aureus* SA1450/94 harbouring pCapAre, which has reconstituted capsule production; **b**) SA1450/94 (control) and **c**) SA1450/94 harbouring pCU1 (vector control).

As capsule production in SA1450/94 might be impaired by the insertion of IS*256* described above, it was attempted to reconstitute CP5 production. In *S. aureus* Newman insertion of Tn*916* into *cap5A1* could be repaired by complementation of *cap5A1* in trans [34]. Therefore, a similar construct (pCapAre) was introduced into SA1450/94, which increased capsule production compared to the parent strain (Figure 8). However, full capsule production was not achieved and the vancomycin MIC of the clone remained unchanged compared to SA1450/94.

Furthermore, a capsule knockout mutant of strain Reynolds had previously been tested against vancomycin, and no differences in susceptibility to vancomycin were recorded [62]. Population analyses in our laboratory confirmed this result (data not shown).

### Effect of capsule material on the susceptibility of staphylococci to vancomycin

In order to test whether capsule material is able to interact with or bind to vancomycin, crude capsular material was prepared from *S. aureus* 137/93G and *S. aureus* NCTC 8325 (negative control; as shown in Figure 6 for *S. aureus* HG001, the strains of this lineage do not produce a capsule unless *cap5E* is repaired). Cell wall teichoic acid that might contaminate the extracts was removed by periodate oxidation. The material was added to MIC determinations using *S. aureus* NCTC8325 and *S. aureus* SG511 as indicator strains in MH medium. There was no significant difference in the MIC values between the extract containing capsular material and the controls for *S. aureus* SG511, however a small effect (0.7 mg/L increase in the MIC) was visible with *S. aureus* NCTC8325 and the extract of *S. aureus* SA137/93G. The test was repeated 8 times with two different preparations of the capsular material; an additional DNase and RNase digest did not influence the result. While we cannot explain this difference, the fact that no increase in the MIC was visible with the more susceptible indicator strain strongly indicated that the type 5 capsular material did not neutralise the effect of vancomycin in this assay. The experiment was repeated with purified CP5 (32 μg/ml) in MIC determinations in MH medium. Both indicator strains did not show any alterations in susceptibility to vancomycin, which confirmed the above result.

## Conclusions

Although an increased transcription of the capsular gene cluster has been observed for several VISA strains, the type 5 capsule does not seem to play a significant role in the resistance mechanism of *S. aureus* 137/93G. It may therefore be assumed that - at least in the strain investigated here - an increased or uniform transcription of the capsule gene cluster is a phenomenon that accompanies vancomycin resistance, perhaps a by-product of a relatively high SigB activity in *S. aureus* 137/93G, indicated by the intense yellow colour of this strain, that might contribute to glycopeptide resistance [50] or an overflow from an activated cell wall metabolism [1], rather than being the cause for vancomycin resistance.

## Competing interests

The authors declare that they have no competing interests.

## Authors’ contributions

AJ designed the study, carried out the microarray and qRT-PCR experiments, performed susceptibility experiments and drafted the manuscript. CS constructed mutants in *S. aureus* SA137/93G, SA1450/94 and *S. aureus* HG001 and performed susceptibility experiments. WS, CW and CG carried out the immunofluorescence visualisation of the capsule polysaccharides, integrated the plasmid pMUTIN4 into the capsule promoter of *S. aureus* Newman and contributed to qRT-PCR experiments. JL gave critical advice for the design of the study, provided capsular antibody, purified CP5, and the Reynolds CP+/CP- strain pair. MT participated in mutant construction. GB conceived the study, participated in its design and drafted the manuscript. All authors read and approved the final manuscript.

## Supplementary Material

Additional file 1 **Gene expression data.pdf. Table S1.** Genes differentially expressed in the hVISA/MRSA strain SA137/93A and the related VSSA/MRSA control strain SA1450/94. **Table S2.** Genes differentially expressed in the VISA/MSSA strain SA137/93G and the VSSA/MRSA control strain SA1450/94. Datasets of 4 microarray experiments (Full Genome Chip *sciTRACER*, Scienion AG, Berlin, Germany) were normalised by applying the LOWESS algorithm and subsequently consolidated using acuity 3.1 software (Axon instruments). Significant changes in gene expression were identified with SAM (significance analysis of microarrays; www-stat.stanford.edu/~tibs/SAM/index.html) software using the one class response type and a false discovery rate of <1%. Click here for file
