# Supplementary material for: Production of capsular polysaccharide does not influence Staphylococcus aureus vancomycin susceptibility
Source: BMC Microbiol. 2013 Mar 22;13:65. doi: 10.1186/1471-2180-13-65 (PMC3617075; doi:10.1186/1471-2180-13-65)
Supplement: Additional file 1 — Gene expression data.pdf. Table S1. Genes differentially expressed in the hVISA/MRSA strain SA137/93A and the related VSSA/MRSA control strain SA1450/94. Table S2. Genes differentially expressed in the VISA/MSSA strain SA137/93G and the VSSA/MRSA control strain SA1450/94. Datasets of 4 microarray experiments (Full Genome Chip sciTRACER, Scienion AG, Berlin, Germany) were normalised by applying the LOWESS algorithm and subsequently consolidated using acuity 3.1 software (Axon instruments). Significant changes in gene expression were identified with SAM (significance analysis of microarrays; www-stat.stanford.edu/~tibs/SAM/index.html) software using the one class response type and a false discovery rate of <1%. [file 1471-2180-13-65-S1.doc]

**Table S1**. Genes differentially expressed in the hVISA/MRSA strain SA137/93A and the related VSSA/MRSA control strain SA1450/94.

| **Gene ID (N315)** | **Component** | **Function** | **Process** | | **Mean of Ratios (>2.0)** |
| --- | --- | --- | --- | --- | --- |
| **Genes with higher expression level in hVISA strain SA137/93A** | | | | | |
| SA0017 | *vicR* (*yycF*) | response regulator | III-5-2 RNA synthesis - Regulation | 11.76 | |
| SA0018 | *vicK* (*yycG*) | two-component sensor histidine kinase | I-3 Sensors (signal transduction) | 12.44 | |
| SA0019 | *yycH** | conserved hypothetical protein, similar to regulator component of YycG (VicK) – *Bacillus subtilis* |  | 11.59 | |
| SA0020 | *yycI** | conserved hypothetical protein, similar to regulator component of YycG (VicK) – *Bacillus subtilis* |  | 3.54 | |
| SA0027 | *repB* | truncated replication protein for plasmid | III-1 DNA replication | 6.01 | |
| SA0093 |  | hypothetical protein |  | 6.75 | |
| SA0095 |  | hypothetical protein |  | 6.97 | |
| SA0096 |  | hypothetical protein |  | 4.72 | |
| SA0097 |  | hypothetical protein, similar to transcription regulator AraC/XylS family | III-5-2 RNA synthesis - Regulation | 4.29 | |
| SA0145 | *capB* | capsular polysaccharide synthesis enzyme Cap5B | IV-1 Adaption to atypical conditions | 4.41 | |
| SA0146 | *capC* | capsular polysaccharide synthesis enzyme Cap5C | IV-1 Adaption to atypical conditions | 4.78 | |
| SA0148 | *capE* | capsular polysaccharide synthesis enzyme Cap5E | IV-1 Adaption to atypical conditions | 4.56 | |
| SA0418 | *cysM* | cysteine synthase homologue | II-2 Metabolism of amino acids and related molecules | 4.83 | |
| SA0846 |  | hypothetical protein, similar to oligopeptide transport system permease protein OppC | I-2 Transport/binding proteins and lipoproteins | 3.23 | |
| SA0847 | *oppD* | oligopeptide transport system ATP-binding protein OppD homologue | I-2 Transport/binding proteins and lipoproteins | 3.01 | |

* Szurmant *et al*., 2005. J Bac. **187**(15):5419-5426; Szurmant *et al*., 2007. J Bac. **189(**8):3280-3289.

**Table S2**. Genes differentially expressed in the VISA/MSSA strain SA137/93G and the VSSA/MRSA control strain SA1450/94.

| **Gene ID (N315)** | **Component** | **Function** | **Process** | **Mean of ratio (>2.0)** |
| --- | --- | --- | --- | --- |
| **I. Genes with higher expression level in VISA strain SA137/93G** | | | | |
| SA0144 | *capA* | capsular polysaccharide synthesis enzyme Cap5A | IV-1 Adaption to atypical conditions | 7.46 |
| SA0145 | *capB* | capsular polysaccharide synthesis enzyme Cap5B | IV-1 Adaption to atypical conditions | 6.58 |
| SA0146 | *capC* | capsular polysaccharide synthesis enzyme Cap5C | IV-1 Adaption to atypical conditions | 8.30 |
| SA0147 | *capD* | capsular polysaccharide synthesis enzyme Cap5D | IV-1 Adaption to atypical conditions | 5.39 |
| SA0148 | *capE* | capsular polysaccharide synthesis enzyme Cap5E | IV-1 Adaption to atypical conditions | 5.16 |
| SA0149 | *capF* | capsular polysaccharide synthesis enzyme Cap5F | IV-1 Adaption to atypical conditions | 7.48 |
| SA0150 | *capG* | capsular polysaccharide synthesis enzyme Cap5G | IV-1 Adaption to atypical conditions | 4.19 |
| SA0151 | *capH* | capsular polysaccharide synthesis enzyme O-acetyl transferase Cap5H | IV-1 Adaption to atypical conditions | 6.23 |
| SA0152 | *capI* | capsular polysaccharide synthesis enzyme Cap5I | IV-1 Adaption to atypical conditions | 8.56 |
| SA0153 | *capJ* | capsular polysaccharide synthesis enzyme Cap5J | IV-1 Adaption to atypical conditions | 5.36 |
| SA0154 | *capK* | capsular polysaccharide synthesis enzyme Cap5K | IV-1 Adaption to atypical conditions | 5.93 |
| SA0155 | *capL* | capsular polysaccharide synthesis enzyme Cap5L | IV-1 Adaption to atypical conditions | 6.79 |
| SA0156 | *capM* | capsular polysaccharide synthesis enzyme Cap5M | IV-1 Adaption to atypical conditions | 5.95 |
| SA0157 | *capN* | capsular polysaccharide synthesis enzyme Cap5N | IV-1 Adaption to atypical conditions | 4.05 |
| SA0158 | *capO* | capsular polysaccharide synthesis enzyme Cap5O | IV-1 Adaption to atypical conditions | 6.59 |
| SA0159 | *capP* | capsular polysaccharide synthesis enzyme Cap5P | IV-1 Adaption to atypical conditions | 5.64 |
| SA0418 | *cysM* | cysteine synthase homologue | II-2 Metabolism of amino acids and related molecules | 3.33 |
| SA0419 | *metB* | cystathionine gamma-synthase | II-2 Metabolism of amino acids and related molecules | 6.39 |
| SA0531 | *proP* | proline/betaine transporter homologue | I-2 Transport/binding proteins and lipoproteins | 2.60 |
| SA0754 |  | hypothetical protein, similar to lactococcal prophage ps3 protein 05 | IV-4 Phage-related functions | 2.15 |
| SA0914 |  | hypothetical protein, similar to chitinase B | IV-7 Miscellaneous | 5.88 |
| SA1898 |  | hypothetical protein, similar to SceD precursor | IV-6 Pathogenic factors (toxins and colonization factors) | 3.37 |
| SA1999 |  | hypothetical protein, similar to regulatory protein. SIR2 family | III-5-2 RNA synthesis – Regulation | 3.41 |
| SA2007 |  | hypothetical protein, similar to alpha-acetolactate decarboxylase | II-1-1 Metabolism of carbohydrates and related molecules – Specific pathways | 2.04 |

**Table S2**. continued

| **Gene ID (N315)** | **Component** | **Function** | **Process** | **Mean of ratio (>2.0)** |
| --- | --- | --- | --- | --- |
| SA2008 | *alsS* | alpha-acetolactate synthase | II-1-1 Metabolism of carbohydrates and related molecules - Specific pathways | 3.22 |
| SA2082 | *ureA* | urease gamma subunit | II-2 Metabolism of amino acids and related molecules | 4.69 |
| SA2083 | *ureB* | urease beta subunit | II-2 Metabolism of amino acids and related molecules | 5.77 |
| SA2084 | *ureC* | urease alpha subunit | II-2 Metabolism of amino acids and related molecules | 6.10 |
| SA2085 | *ureE* | urease accessory protein UreE | II-2 Metabolism of amino acids and related molecules | 4.11 |
| SA2086 | *ureF* | urease accessory protein UreF | II-2 Metabolism of amino acids and related molecules | 7.55 |
| SA2087 | *ureG* | urease accessory protein UreG | II-2 Metabolism of amino acids and related molecules | 5.22 |
| SA2088 | *ureD* | urease accessory protein UreD | II-2 Metabolism of amino acids and related molecules | 3.65 |
| SA2097 |  | hypothetical protein, similar to secretory antigen precursor SsaA | IV-6 Pathogenic factors (toxins and colonization factors) | 3.04 |
| SA2226 | *truncated-SA* | truncated hypothetical protein, similar to D-serine/D-alanine/glycine transporter | I-2 Transport/binding proteins and lipoproteins | 2.59 |
| SA2262 |  | conserved hypothetical protein |  | 2.14 |
| SA2312 | *ddh* | D-specific D-2-hydroxyacid dehydrogenase | II-1-1 Metabolism of carbohydrates and related molecules - Specific pathways | 3.06 |
| SA2320 |  | hypothetical protein, similar to regulatory protein pfoR | III-5-2 RNA synthesis - Regulation | 2.53 |
| SA2329 |  | conserved hypothetical protein |  | 2.94 |
| SA2355 |  | conserved hypothetical protein |  | 2.28 |
| SA2367 |  | conserved hypothetical protein |  | 2.12 |
| SA2384 | *ermA* | rRNA methylase Erm(A) | III-6 RNA modification | 2.04 |
| SA2403 |  | conserved hypothetical protein |  | 2.11 |
| SA2459 | *icaA* | intercellular adhesion protein A | IV-6 Pathogenic factors (toxins and colonization factors) | 4.34 |
| SA2460 | *icaD* | intercellular adhesion protein D | IV-6 Pathogenic factors (toxins and colonization factors) | 2.22 |
| **II. Genes with higher expression level in VSSA strain SA1450/94** | | | | |
| SA0025 |  | hypothetical protein |  | 5.19 |
| SA0133 | *dra* | deoxyribose-phosphate aldolase | II-3 Metabolism of nucleotides and nucleic acids | 3.89 |
| SA0143 | *adhE* | alcohol-acetaldehyde dehydrogenase | II-1-1 Metabolism of carbohydrates and related molecules - Specific pathways | 4.22 |
| SA0183 | *glcA* | PTS enzyme II (EC 2.7.1.69), glucose-specific, factor IIA homologue | I-2 Transport/binding proteins and lipoproteins | 2.43 |
| SA0218 | *pflB* | formate acetyltransferase | II-1-1 Metabolism of carbohydrates and related molecules - Specific pathways | 3.98 |
| SA0219 | *pflA* | formate acetyltransferase activating enzyme | II-1-1 Metabolism of carbohydrates and related molecules - Specific pathways | 5.15 |

**Table S2**. continued

| **Gene ID (N315)** | **Component** | **Function** | **Process** | **Mean of ratio (>2.0)** |
| --- | --- | --- | --- | --- |
| SA0242 |  | hypothetical protein, similar to xylitol dehydrogenase | II-1-1 Metabolism of carbohydrates and related molecules - Specific pathways | 2.72 |
| SA0294 |  | hypothetical protein, similar to branched-chain amino acid uptake carrier | I-2 Transport/binding proteins and lipoproteins | 4.29 |
| SA0337 |  | hypothetical protein, similar to transcriptional repressor | III-5-2 RNA synthesis - Regulation | 4.37 |
| SA0339 |  | hypothetical protein, similar to ABC transporter ATP-binding protein | I-2 Transport/binding proteins and lipoproteins | 5.10 |
| SA0340 |  | conserved hypothetical protein |  | 2.84 |
| SA0397 | *lpl2* | hypothetical protein [Pathogenicity island SaPIn2] |  | 2.46 |
| SA0406 |  | hypothetical protein |  | 3.44 |
| SA0407 |  | conserved hypothetical protein |  | 4.55 |
| SA0452 | *veg* | VEG protein homologue |  | 2.59 |
| SA0453 |  | hypothetical protein, similar to 4-diphosphocytidyl-2-C-methyl-D-erythritol kinase | II-3 Metabolism of nucleotides and nucleic acids | 2.41 |
| SA0589 |  | hypothetical protein, similar to ABC transporter ATP-binding protein | I-2 Transport/binding proteins and lipoproteins | 3.04 |
| SA0677 |  | hypothetical protein, similar to choline transport ATP-binding protein | I-2 Transport/binding proteins and lipoproteins | 2.33 |
| SA0678 |  | hypothetical protein, similar to choline transporter | I-2 Transport/binding proteins and lipoproteins | 2.49 |
| SA0707 |  | conserved hypothetical protein |  | 2.54 |
| SA0746 |  | staphylococcal nuclease | IV-6 Pathogenic factors (toxins and colonization factors) | 2.26 |
| SA0747 | *cspC* | cold-shock protein C | IV-1 Adaption to atypical conditions | 2.59 |
| SA0801 |  | conserved hypothetical protein |  | 13.69 |
| SA0835 | *clpB* | ClpB chaperone homologue | IV-1 Adaption to atypical conditions | 3.30 |
| SA0890 |  | conserved hypothetical protein |  | 2.09 |
| SA1002 |  | hypothetical protein |  | 2.67 |
| SA1003 |  | hypothetical protein, similar to fibrinogen-binding protein | IV-6 Pathogenic factors (toxins and colonization factors) | 2.07 |
| SA1072 | *plsX* | fatty acid/phospholipids synthesis protein | II-4 Metabolism of lipids | 2.70 |
| SA1074 | *fabG* | 3-oxoacyl-reductase, acyl-carrier protein | II-4 Metabolism of lipids | 2.19 |
| SA1165 | *thrC* | threonine synthase | II-2 Metabolism of amino acids and related molecules | 2.16 |
| SA1166 | *thrB* | homoserine kinase homologue | II-2 Metabolism of amino acids and related molecules | 2.27 |
| SA1174 | *lexA* | SOS regulatory LexA protein | III-5-2 RNA synthesis – Regulation | 2.40 |
| SA1243 |  | ABC transporter homologue | I-2 Transport/binding proteins and lipoproteins | 2.19 |
| SA1269 |  | Blt-like protein | I-2 Transport/binding proteins and lipoproteins | 2.38 |
| SA1270 |  | hypothetical protein, similar to amino acid permease | I-2 Transport/binding proteins and lipoproteins | 2.77 |
| SA1272 |  | alanine dehydrogenase | II-2 Metabolism of amino acids and related molecules | 2.76 |
| SA1401 |  | conserved hypothetical protein |  | 2.45 |
| SA1402 |  | conserved hypothetical protein |  | 2.52 |
| SA1403 |  | conserved hypothetical protein |  | 2.08 |
| SA1465 | *tgt* | tRNA-guanine transglycosylase | III-6 RNA modification | 2.43 |

**Table S2**. continued

| **Gene ID (N315)** | **Component** | **Function** | **Process** | **Mean of ratio (>2.0)** |
| --- | --- | --- | --- | --- |
| SA1506 | *thrS* | threonyl-tRNA synthetase 1 | III-7-2 Protein synthesis – Aminoacyl-tRNA synthases | 2.83 |
| SA1520 | *pykA* | pyruvate kinase | II-1-2 Metabolism of carbohydrates and related molecules – Main glycolytic pathway | 2.83 |
| SA1532 |  | conserved hypothetical protein |  | 2.45 |
| SA1553 | *fhs* | formyltetrahydrofolate synthetase | II-1-1 Metabolism of carbohydrates and related molecules – Specific pathways | 2.65 |
| SA1602 |  | hypothetical protein |  | 13.21 |
| SA1609 | *pckA* | phosphoenolpyruvate carboxykinase | II-1-2 Metabolism of carbohydrates and related molecules – Main glycolytic pathway | 2.90 |
| SA1756 | *lytA* | truncated amidase [Bacteriophage phiN315] | IV-4 Phage-related functions | 2.04 |
| SA1757 | *lytA* | truncated amidase [Bacteriophage phiN315] | IV-4 Phage-related functions | 2.18 |
| SA1812 |  | hypothetical protein, similar to synergohymenotropic toxin precursor – *Staphylococcus intermedius* | IV-6 Pathogenic factors (toxins and colonization factors) | 2.17 |
| SA1813 |  | hypothetical protein, similar to leukocidin chain LukM precursor | IV-6 Pathogenic factors (toxins and colonization factors) | 3.22 |
| SA1821 |  | hypothetical protein [Pathogenicity island SaPIn1] |  | 2.17 |
| SA1836 | *groEL* | GroEL protein | III-9 Protein folding | 3.75 |
| SA1875 | *dpj* | holo-ACP synthase | III-8 Protein modification | 2.65 |
| SA1876 |  | conserved hypothetical protein |  | 2.35 |
| SA1939 |  | deoxyribose-phosphate aldolase | II-3 Metabolism of nucleotides and nucleic acids | 4.11 |
| SA2051 | *topB* | DNA topoisomerase III TopB | III-4 DNA packaging and segregation | 3.19 |
| SA2091 |  | hypothetical protein |  | 2.36 |
| SA2092 |  | hypothetical protein, similar to transcription regulator | III-5-2 RNA synthesis – Regulation | 2.69 |
| SA2134 |  | hypothetical protein, similar to DNA-3-methyladenine glycosidase | III-2 DNA replication/modification and repair | 3.18 |
| SA2156 |  | L-lactate permease LctP homolog ue | I-2 Transport/binding proteins and lipoproteins | 3.02 |
| SA2176 | *narK* | nitrite extrusion protein | I-2 Transport/binding proteins and lipoproteins | 3.30 |
| SA2183 |  | hypothetical protein, similar to nitrate reductase delta chain | I-4 Membrane bioenergetics (electron transport chain and ATP synthase) | 2.16 |
| SA2184 | *narH* | nitrate reductase beta chain NarH | I-4 Membrane bioenergetics (electron transport chain and ATP synthase) | 2.25 |
| SA2185 | *narG* | respiratory nitrate reductase alpha chain | I-4 Membrane bioenergetics (electron transport chain and ATP synthase) | 3.89 |
| SA2192 |  | hypothetical protein |  | 3.05 |
| SA2200 |  | hypothetical protein, similar to ABC transporter, ATP binding subunit | I-2 Transport/binding proteins and lipoproteins | 2.28 |
| SA2201 |  | hypothetical protein, similar to ABC transporter, permease protein | I-2 Transport/binding proteins and lipoproteins | 2.24 |
| SA2202 |  | hypothetical protein, similar to ABC transporter, periplasmic amino acid-binding protein | I-2 Transport/binding proteins and lipoproteins | 2.29 |
| SA2208 | *hlgC* | gamma-hemolysin component C | IV-6 Pathogenic factors (toxins and colonization factors) | 2.63 |
| SA2209 | *hlgB* | gamma-hemolysin component B | IV-6 Pathogenic factors (toxins and colonization factors) | 2.42 |
| SA2251 | *opp-1F* | oligopeptide transporter putative ATPase domain | I-2 Transport/binding proteins and lipoproteins | 2.70 |

**Table S2**. continued

| **Gene ID (N315)** | **Component** | **Function** | **Process** | **Mean of ratio (>2.0)** |
| --- | --- | --- | --- | --- |
| SA2252 | *opp-1D* | oligopeptide transporter putative ATPase domain | I-2 Transport/binding proteins and lipoproteins | 3.05 |
| SA2255 | *opp-1A* | oligopeptide transporter putative substrate binding domain | I-2 Transport/binding proteins and lipoproteins | 2.88 |
| SA2268 |  | hypothetical protein |  | 3.00 |
| SA2302 |  | hypothetical protein, similar to ABC transporter | I-2 Transport/binding proteins and lipoproteins | 2.34 |
| SA2303 |  | hypothetical protein, similar to membrane spanning protein | I-2 Transport/binding proteins and lipoproteins | 3.19 |
| SA2346 |  | hypothetical protein, similar to D-specific D-2-hydroxyacid dehydrogenase Ddh homologue | II-1-1 Metabolism of carbohydrates and related molecules - Specific pathways | 2.81 |
| SA2347 |  | hypothetical protein, similar to aspartate aminotransferase | II-2 Metabolism of amino acids and related molecules | 2.16 |
| SA2357 |  | hypothetical protein, similar to regulatory protein (PfoS/R) | III-5-2 RNA synthesis - Regulation | 2.05 |
| SAS014 |  | hypothetical protein |  | 2.55 |
| SAS022 | *truncated-SA* | truncated conserved hypothetical protein |  | 3.05 |
